# An X-band Bi-Directional Transmit/Receive Module for a Phased Array System in 65-nm CMOS

**DOI:** 10.3390/s18082569

**Published:** 2018-08-06

**Authors:** Van-Viet Nguyen, Hyohyun Nam, Young Joe Choe, Bok-Hyung Lee, Jung-Dong Park

**Affiliations:** 1 Division of Electronics and Electrical Engineering, Dongguk University, Seoul 04620, Korea; vietbk10@dongguk.edu (V.-V.N.); kahn0217@dongguk.edu (H.N.); youngjoechoe@dongguk.edu (Y.J.C.); 2 Yongin Research Institute, Hanwha Systems, Gyeonggi-do 17121, Korea; bokhyung1228.lee@hanwha.com

**Keywords:** radar-based sensors, phased array antenna, phase shifter, attenuator, bi-directional gain amplifier, T/R module

## Abstract

We present an X-band bi-directional transmit/receive module (TRM) for a phased array system utilized in radar-based sensor systems. The proposed module, comprising a 6-bit phase shifter, a 6-bit digital step attenuator, and bi-directional gain amplifiers, is fabricated using 65-nm CMOS technology. By constructing passive networks in the phase-shifter and the variable attenuator, the implemented TRM provides amplitude and phase control with 360° phase coverage and 5.625° as the minimum step size while the attenuation range varies from 0 to 31.5 dB with a step size of 0.5 dB. The fabricated T/R module in all of the phase shift states had RMS phase errors of less than 4° and an RMS amplitude error of less than 0.93 dB at 9–11 GHz. The output 1dB gain compression point (OP1dB) of the chip was 5.13 dBm at 10 GHz. The circuit occupies 3.92 × 2.44 mm^2^ of the chip area and consumes 170 mW of DC power.

## 1. Introduction

Active Electronically Scanned Arrays (AESAs) are gradually replacing mechanical scanning radar in radar systems today and will continue to do so in the future [1]. Transmit/receive modules (TRMs) play the most critical role in active phase array systems employed in many radar and electronic warfare applications [2,3]. Currently, most commercialized TRMs are GaAs pseudomorphic High Electron Mobility Transistor (pHEMT) devices [4,5]. Recently, TR chipsets have also been reportedly used in the SiGe BiCMOS process [6,7,8,9,10]. Even though high RF performances are achievable, relatively high power consumption and relatively higher product cost make them less attractive. Along with the advance in chip packaging, 3D-RF system-in-package (SiP) technology brings the advantages of compact size, electromagnetic isolation, and effective interconnection as presented in [11,12,13,14]. Thousands of TRMs may be needed to realize an AESA system, thus reducing the cost per each TRM has a significant meaning in the cost reduction of an array system for various low-cost commercial applications. Advanced innovations in the standard CMOS process can bring benefits of lower cost, higher integration, and the low power consumption as well [15,16,17,18]. Typically, CMOS T/R modules must perform the agile control of gain, amplitude, and phase to steer the antenna beam accurately [19,20]. Therefore, they consist of a phase shifter and attenuator blocks, and a bi-directional gain amplifier (BDGA) to compensate for the insertion loss due to the CMOS switches in the passive control blocks [21,22]. Realizing TRMs in standard CMOS technology offers many advantages, but is still a challenging task, especially in achieving low-loss switches with MOS. Much effort has been made to improve the performance of CMOS switches by minimizing or maximizing the substrate resistance [23,24,25] and by using the body-floating approach [26,27].

In the proposed TRM, the double-well body-floating technique is used in the design of single-pole-double-through (SPDT), and double-pole-double-through (DPDT) switches employed in the phase shifter and attenuator to improve the power handling capability [28,29]. The proposed CMOS TRM is designed for the next-generation weather radar system where low-cost and low-power consumption are essential requirements in implementing the AESA system [30]. The designed TRM has been fabricated in 65-nm CMOS technology with a 1.2 V supply. The detailed design procedure of the X-band CMOS TRM with corresponding simulation results is discussed in detail in Section 2, and the measurement setup and experimental results of the implemented TRM in a 65-nm CMOS are presented in Section 3, which is followed by the conclusions on the study.

## 2. The Design of the X-band TRM

**Figure 1:**
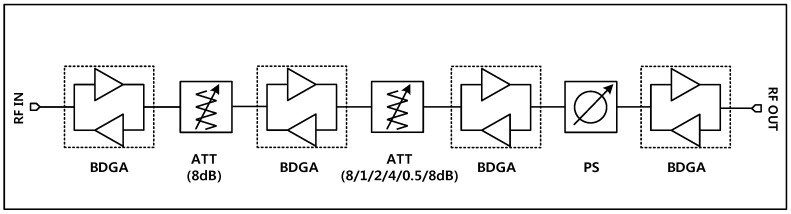
A block diagram of the proposed X-band TRM.

Figure 1 illustrates the configuration of the proposed TRM. The structure consists of a 6-bit phase shifter block, a 6-bit digital attenuator, and BDGAs for loss compensation. The phase shifter block is placed between two BDGAs to achieve better input and output return losses, thus resulting in a reduction in phase and amplitude variations in all of the phase shift states. The attenuator block is made up of attenuation units interspersed with BDGA blocks to diminish the loading effect which may cause unusual attenuation steps. There are four BDGAs employed in the proposed structure to compensate for the insertion loss of the attenuator and phase shifter blocks and also to provide a specific gain for the entire system.

### 2.1. The 6-Bit Phase Shifter

The phase shifter is a crucial element in phased array antenna systems. Its phase shifting capability should be agile enough to steer the main lobe of the arrayed antenna precisely. In CMOS design, the use of high-pass/low-pass (HP/LP) phase shifter topology is advantageous because of its advantages of power, a digital control mechanism, broadband operation, and less reliance on the RF performance of the active device. The basic concept of the HP/LP phase shifter is to use its phase leading and lagging characteristics from the pole and zero. An HP filter comprising series capacitors and shunt inductors provides phase leading while an LP filter composed of series inductors and shunt capacitors generates phase lagging. By applying a switching mechanism between the LP and HP sections, the network can function as a phase shifter with wideband performance.

**Figure 2:**
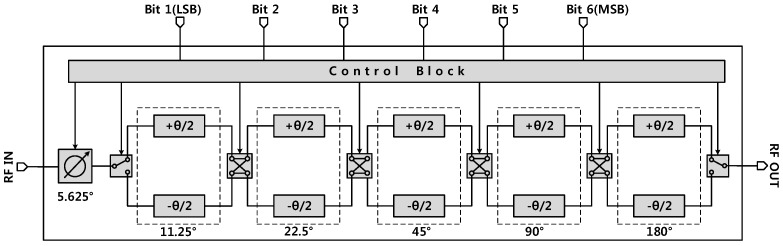
A block diagram of the proposed 6-bit phase shifter.

Figure 2 shows the block diagram of the designed phase shifter used in this work. The phase shifter consists of two SPDT switches, four DPDT switches, phase shifting elements, and a digital controller. Six bits of the digital control signals are input to the digital decoder through the SPI scan chain, which controls the corresponding switches of the phase shifter. The proposed phase shifter covers a range of 360° with a least significant bit (LSB) of 5.625°. Design optimization was performed on the HP/LP filter networks, and the SPDT and DPDT switch separately.

#### 2.1.1. The phase Shifting Elements

First, the design and optimization of each phase shift element are performed. Since an HP filter provides phase leading while an LP filter generates phase lagging, each path is set to a half of the desired amount of phase shift such that the phase difference between the two branches becomes the expected phase shift. The design equations of *L* and *C* elements are summarized in Table 1.

It should be noted that we should avoid implementing phase leading blocks at 11.25°, 22.5°, and 45° in the HP filter topology since it requires excessively large inductances in the realization on the chip. To avoid a large inductance, we use a bandpass (BP) filter structure for the leading phase branches of the above-mentioned phase-shift elements:

(1) $$L=\frac{1-\tan\phi_{21}\cdot 2Z_{0}\omega C}{2C\omega^{2}}.$$

Equation (1) shows the relationship between the values of the *L* and *C* elements with the phase characteristic of the network transfer function. A summary of the realized values of the passive components used in the phase shifter design is contained in Table 2.

**Figure 3:**
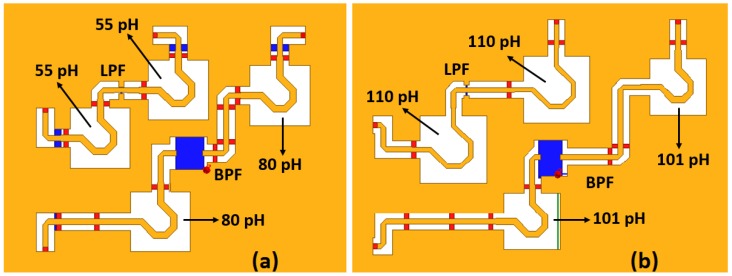
The passive structure of the 11.25 (**a**) and 22.5 (**b**) phase shift units simulated with HFSS.

The inductors and capacitors in the phase shifting cells are fine-tuned to achieve precise phase shift levels. To reduce the chip area occupation, spiral inductors with a top metal layer of aluminum are implemented while metal insulator metal (MIM) capacitors available in the process design kit (PDK) are utilized. All of the passive structures of the phase shift units are simulated with a full-wave electromagnetic (EM) simulator, HFSS, as shown in Figure 3 for 11.25 and 22.5 phase shift units.

#### 2.1.2. The SPDT and DPDT Switches

**Figure 4:**
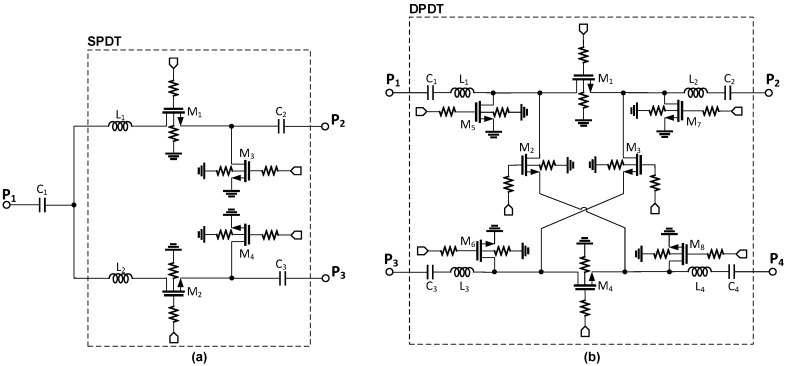
Circuit schematics of the SPDT (**a**) and DPDT (**b**) switches.

To perform phase shifting functionality covering 360° of the phase control with a step of 5.625°, we need to implement a low-loss switching mechanism in the phase-shifter block at the X-band. Switches enable us to configure the signal path through phase shifting elements to produce the desired phase shift levels. The proposed phase shifter employs two SPDT and four DPDT switches whose circuit schematics are depicted in Figure 4a,b, respectively. For the SPDT switches, series transistors *M*_1_ and *M*_2_ perform the main switching function, while shunt transistors *M*_3_ and *M*_4_ are added to improve the isolation between the different paths. The operations of the series and shunt transistors in the same branch are complementary, and only one path is activated at a time.

**Figure 5:**
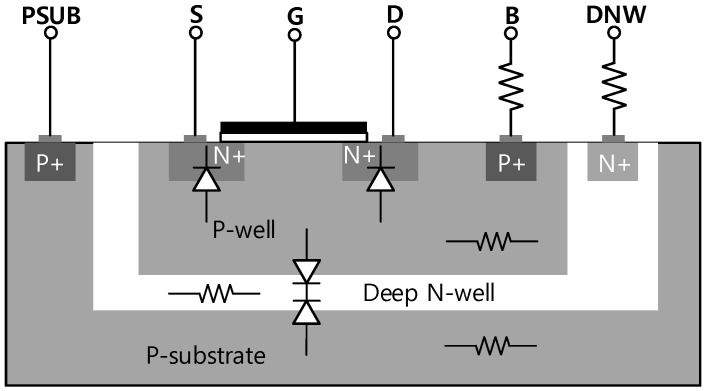
A schematic illustration of the double-well body-floating technique.

In the design of the SPDT and DPDT switches, the gate control terminals are biased through a large resistor *R*_G_ to reduce fluctuations in the *V*_GD_ and *V*_GS_ of the transistors due to voltage swings at the drain and source terminals. This configuration maintains the ON resistance of the transistor unchanged and avoids an excessive voltage across the gate dielectric which might otherwise lead to a breakdown issue. Due to the conductive silicon substrate, MOSFETs used as switches in SPDT and DPDT induce a relatively high insertion loss. To alleviate the adverse effect of the lossy substrate, we applied a double-well body-floating technique which also enhances the power handling capability of the circuit. In the triple-well CMOS process, the local P-well and global P-substrate are separated by a deep N-well layer which introduces two new diodes, the diode between the P-well and the deep N-well, and the diode between the deep N-well and the P-substrate. A strong input signal can make source-body and drain-body diodes turn-on unintentionally, which might cause linearity degradation, and so the body of a MOSFET device should be kept as a high impedance node to prevent this phenomenon. Afterward, the body voltage is bootstrapped to the voltage swing of the input signal. This body-floating technique can be readily realizable by biasing the body of the P-well through a large resistor. However, maintaining the P-well at a high-impedance may result in another accidental turn-on with the diode between the P-well and the deep N-well layer. Thus, the deep N-well should also be biased through a large resistor so that it effectively floats in the RF frequency. Figure 5 shows a simplified cross-sectional view of an NMOS in a triple-well with resistors to achieve high-impedance nodes.

Table 3 provides the circuit parameters of the devices used in the SPDT and DPDT switch designs. At 10 GHz, the simulated insertion losses of the SPDT and DPDT switches are around 1.9 dB and 2.5 dB, respectively, and the input/output return loss is better than 12 dB over the frequency range of 8–12 GHz. Since it is implemented with passive devices only, the proposed phase shifter can serve in both the transmit and receive operation modes.

**Figure 6:**
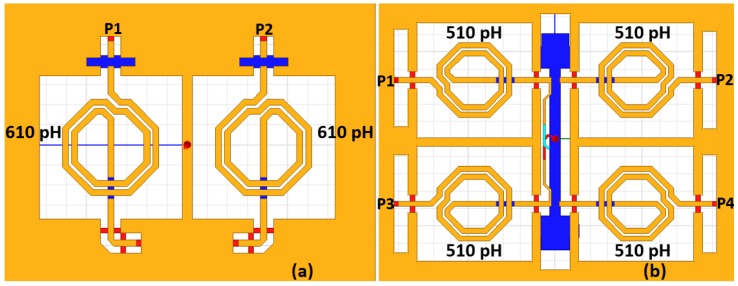
The passive structure of the SPDT (**a**) and DPDT (**b**) switches simulated with HFSS.

All of the passive structures in the SPDT/DPDT switches were simulated with HFSS to consider the coupling effects of each inductor, as presented in Figure 6.

**Figure 7:**
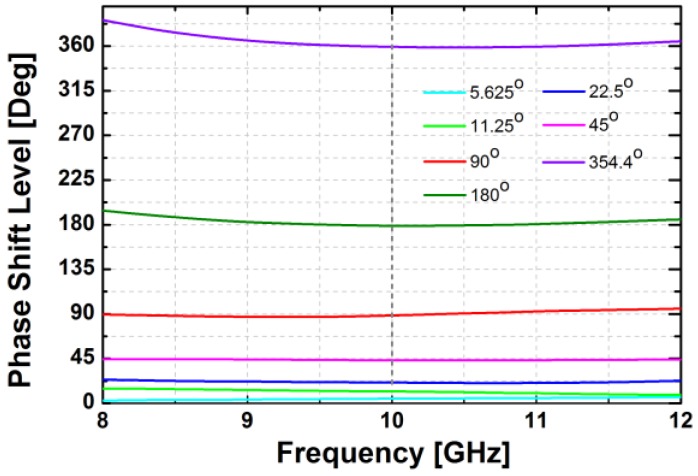
Simulated relative phase shift levels in the main states of the phase shifter block.

**Figure 8:**
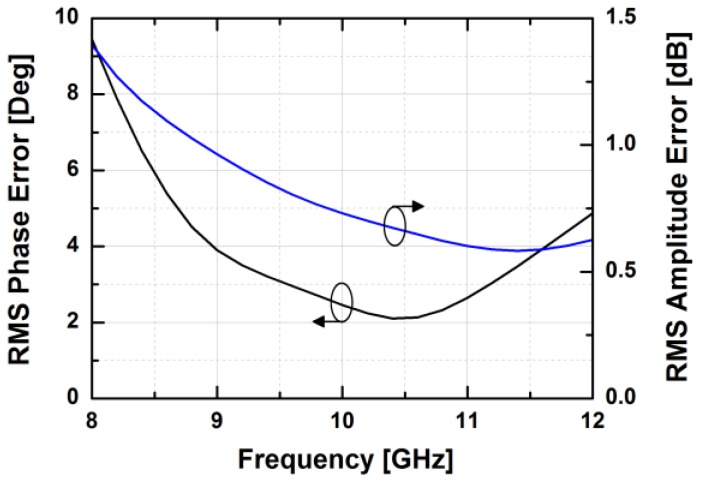
The RMS phase and amplitude errors in all of the phase shifter’s phase shift states.

The simulated insertion loss of the designed phase shifter with zero phase shifting is less than 16 dB at 10 GHz. Figure 7 demonstrates the phase responses of the phase shifter in the main states. The proposed phase shifter provides a phase shift of 360° with an LSB of 5.625°. The simulated RMS phase error is less than 4° while the systematic RMS amplitude error is less than 1 dB at frequencies from 9 to 11 GHz, as presented in Figure 8. Owing to the HP/LP topology with on-chip passive elements, the proposed phase shifter does not consume DC power, except for the leakage current from the MOS switches.

### 2.2. The Attenuator

**Figure 9:**
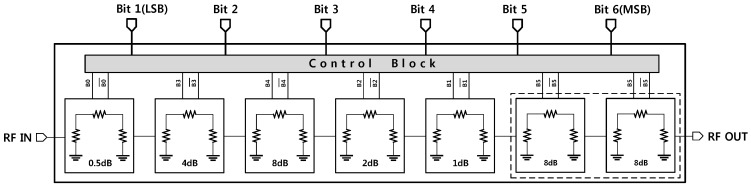
A block diagram of the 6-bit attenuator.

Figure 9 presents the proposed structure of the digitally controlled attenuator. A 6-bit CMOS digital attenuator was designed with a resistive Pi-type structure instead of a T-type in consideration of the range of resistance, and it is possible to fabricate it in practice. The proposed circuit covers the range of 0–31.5 dB of attenuation with a step of 0.5 dB. The whole of the 16 dB cell is separated into two 8-dB cells for optimal performance over the designated frequency range.

**Figure 10:**
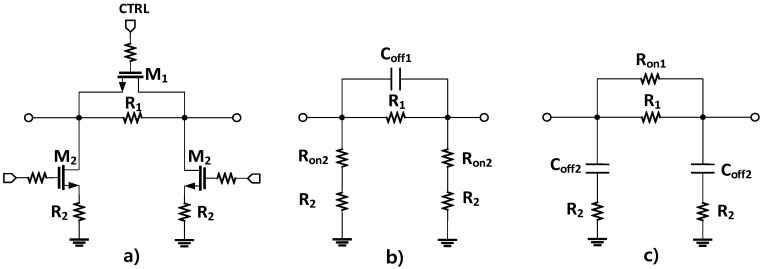
A schematic of the Pi-type resistive attenuator cell (**a**) and simplified models of it in the OFF (**b**) and ON (**c**) states.

In the designed attenuator, the relative attenuation level is obtained by taking the amplitude difference between the attenuation state and the reference state, which are controlled by single NMOS switches determining the performance of the digital step attenuator. A single NMOS switch can be approximately modeled as a channel resistor *R*_ON_ in the ON state and an equivalent parasitic capacitor *C*_OFF_ in the OFF state. By neglecting the parasitic body capacitance and the series parasitic inductance, the schematic and equivalent circuits of the attenuation and reference states are as shown in Figure 10. Table 4 provides the circuit parameters of the devices used in the attenuator design.

**Figure 11:**
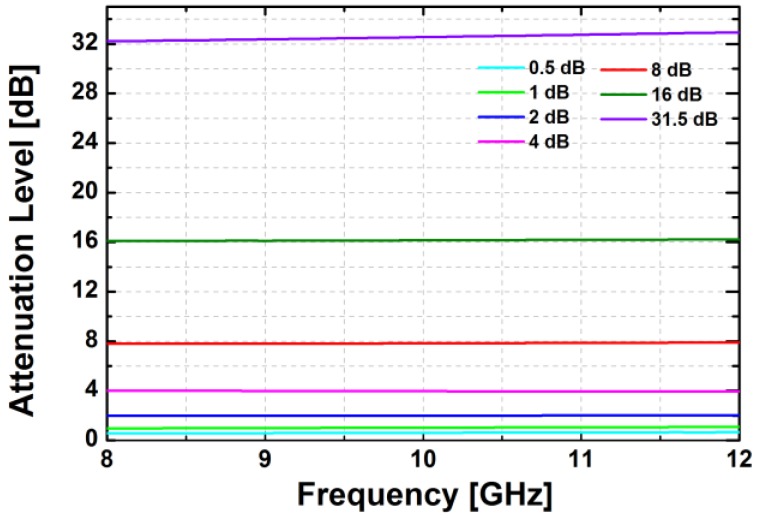
The simulated relative attenuation levels in the main states of the attenuator block.

**Figure 12:**
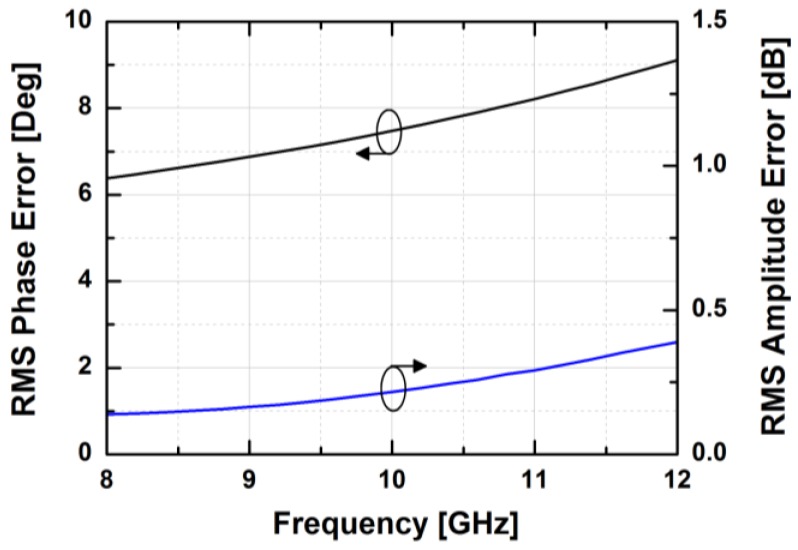
The RMS phase and amplitude errors in all of the attenuator’s attenuation states.

The simulated insertion loss of the attenuator block in the zero attenuation level is less than 10 dB at 10 GHz. As shown in Figure 11, the designed attenuator provides 31.5 dB of the maximum attenuation level with a step size of 0.5 dB in the simulation. In the frequency range of 9–11 GHz, the simulated RMS amplitude error is less than 0.5 dB while the simulated RMS phase error is less than 8.2°, as depicted in Figure 12.

### 2.3. The Bi-Directional Gain Amplifier (BDGA)

**Figure 13:**
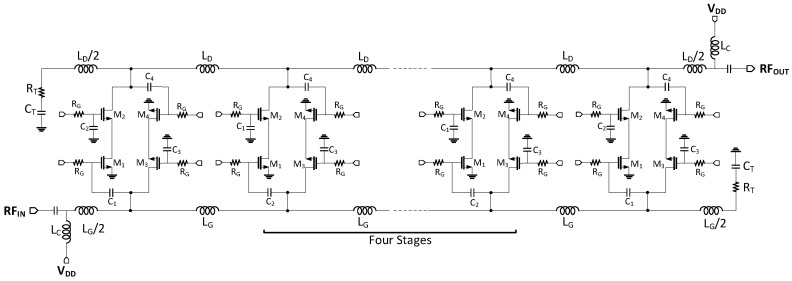
A circuit schematic of the proposed BDGA with six stages of gain cells.

The BDGA is used to provide the designated gain to the TRM. Each distributed stage of the BDGA is made up of two cascode transistor pairs to provide the bi-directional operation for transmitting and receiving RF signals. Based on the control status, each branch takes responsibility for amplifying signals in both the forward and reverse modes. Figure 13 shows a circuit schematic of the proposed BDGA.

The BDGA consists of multiple stages of cascode transistor pairs (*M*_1_~*M*_4_), gate and drain inductors (*L*_G_ and *L*_D_), 50 Ω termination resistors (*R*_T_), and RF choke inductors (*L*_C_). The cascode configuration provides several benefits including a better high-frequency response, high bandwidth, and simpler biasing. The mechanism to control the amplification direction of the BDGA is set by activating one of the cascode transistor pairs, which can be done by switching the bias voltages applied to the gate terminal of the transistors. For instance, in the forward operation, transistors *M*_1_ and *M*_2_ are ON in the saturation region and transistors *M*_3_ and *M*_4_ are OFF. The shunt capacitances at the input comprise the gate capacitance of *M*_1_ in the saturation region and the drain capacitance of *M*_3_ in the OFF region, which along with gate inductances *L*_G_/2, form the artificial gate transmission line. By appropriately choosing the MOS sizes and the inductor values, its characteristic impedance is approximated by $Z_{0}=\sqrt{L_{G}/C_{G}}=\sqrt{L_{D}/C_{D}}$, which is designed to achieve 50 Ω. As a result, the circuit provides a wideband frequency response with better input and output return losses.

To supply the bias current to the reverse and forward amplifiers, two V_DD_ are connected at the ends of the amplifier through choke inductors (*L*_C_). The capacitor at the gate of the input transistor isolates its gate bias voltage from the V_DD_. All bias voltages are provided through 30 kΩ resistors, as shown in Figure 13. All of the parasitics and coupling of the passive elements were considered by performing a 3-D EM simulation with HFSS.

**Figure 14:**
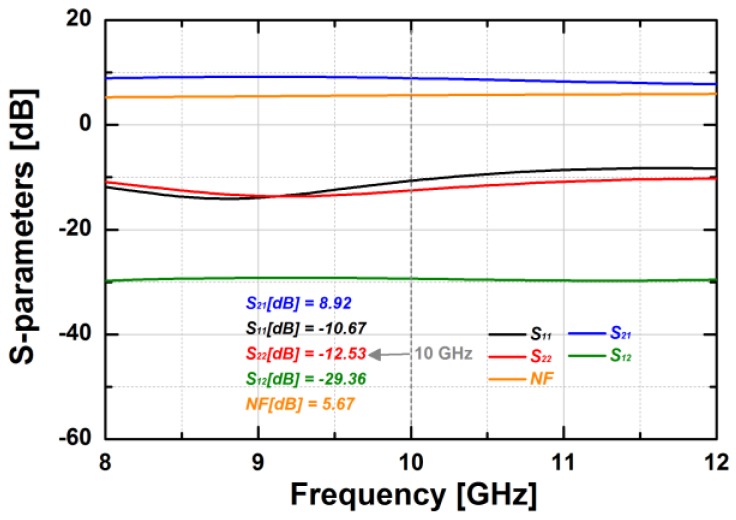
The simulated S-parameters of the BDGA.

**Figure 15:**
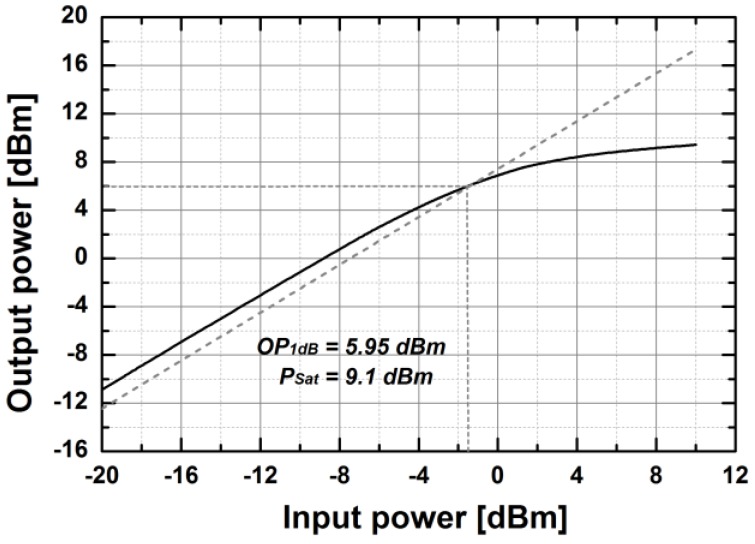
The simulated input/output power characteristics of the BDGA.

**Figure 16:**
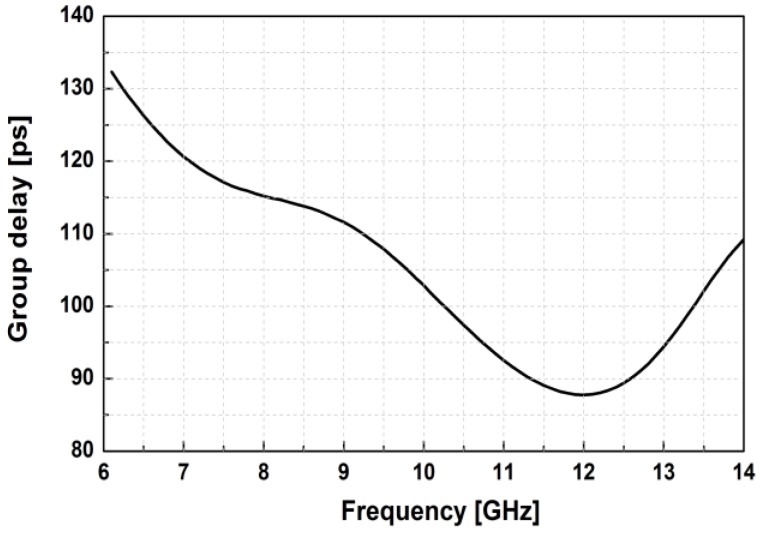
The group delay of the BDGA at frequencies from 6–14 GHz.

Figure 14 shows the simulated S-parameters of the BDGA block. The simulated gain of the BDGA is higher than 8.5 at 10 GHz, and the input/output return losses are better than 10 dB at frequencies from 9 to 11 GHz. The simulated noise figure (NF) is less than 6 dB at 50 °C. The total DC current consumption is 24 mA from a 1.2 V supply voltage. The reverse operation performances are almost the same as the forward one owing to the symmetrical layout. The simulated OP1dB is 5.95 dBm, and the saturated output power is 9.1 dBm at 10 GHz, as given in Figure 15. As shown in Figure 16, the simulated group delay of the BDGA is less than 110 ps from 9 to 11 GHz.

## 3. Measurement Results

**Figure 17:**
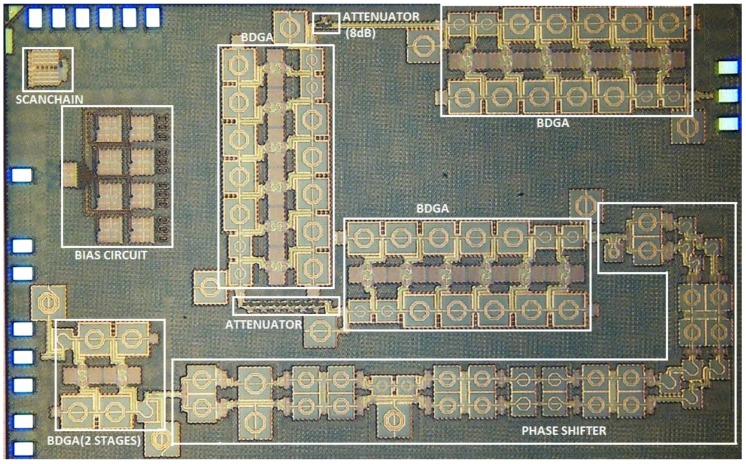
A microphotograph of the X-band bi-directional transmit/receive module.

Figure 17 presents a microphotograph of the implemented X-band bi-directional T/R module fabricated in 65-nm CMOS technology. The total area of the chip including pads is 3.92 × 2.44 mm^2^. All of the measurements were carried out with on-chip probing.

**Figure 18:**
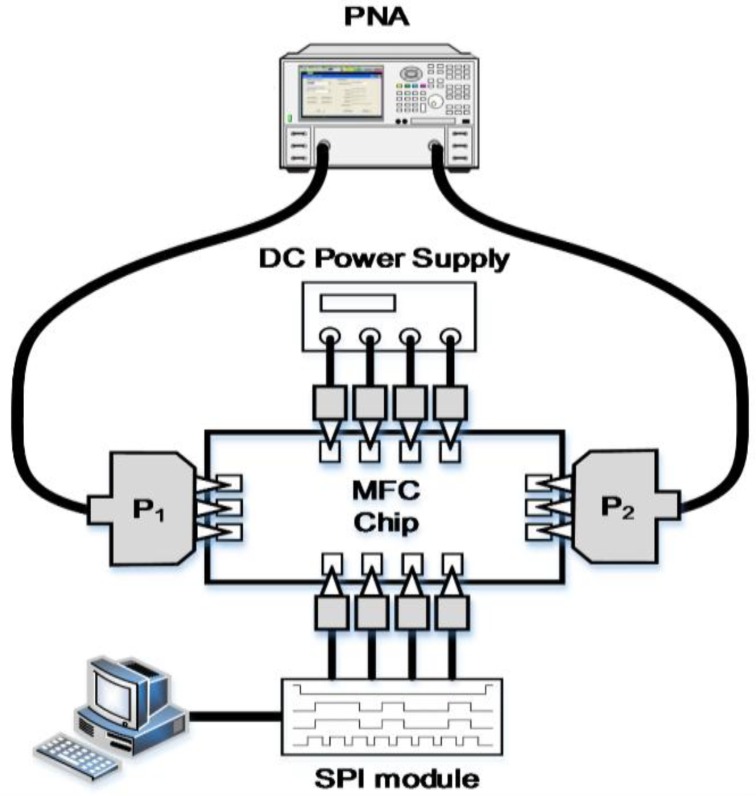
The block diagram of S-parameters, phase and attenuation response measurement setup as a function of the control bits.

**Figure 19:**
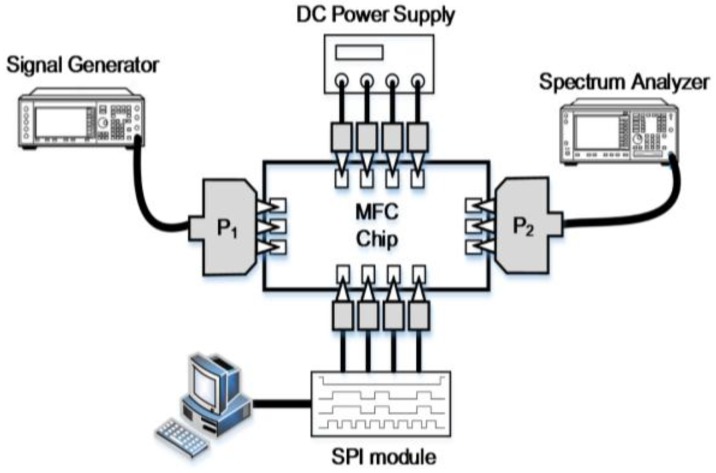
The block diagram of the output power and the gain compression measurement setup as a function of the input power (AM-AM).

Figure 18 illustrates the block diagram of measuring S-parameters, phase and attenuation response as a function of the control bits. The measurement setup for the output power and the gain compression measurement setup as a function of the input power (AM-AM) is presented in Figure 19. A 64-bit SPI scan-chain has been integrated into the TRM and interfaced with an external laptop computer not only to control the phase and attenuation levels but also to correct the bias of the internal blocks for the optimal performance of the TRM. The equipment used for measurement is as follows: Keysight DSO-X 6002A digital oscilloscope to check the SPI signal, Agilent E4407B spectrum analyzer, Agilent 83623B signal generator, Keysight N5224A network analyzer, and Agilent B2902A power supply.

**Figure 20:**
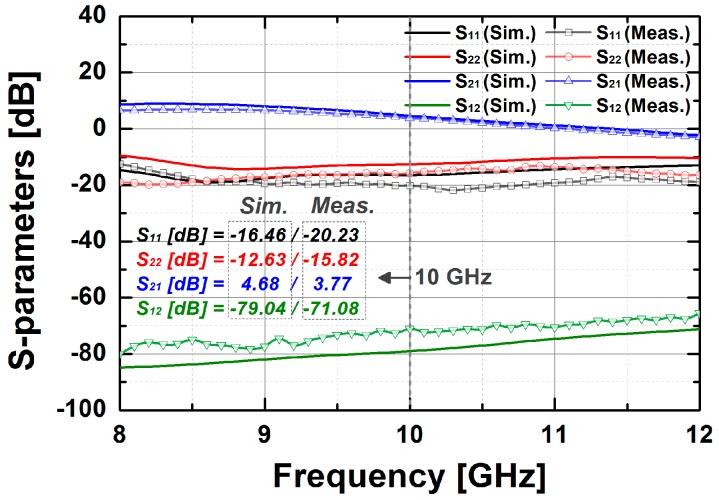
The measured S-parameters of the implemented TRM.

**Figure 21:**
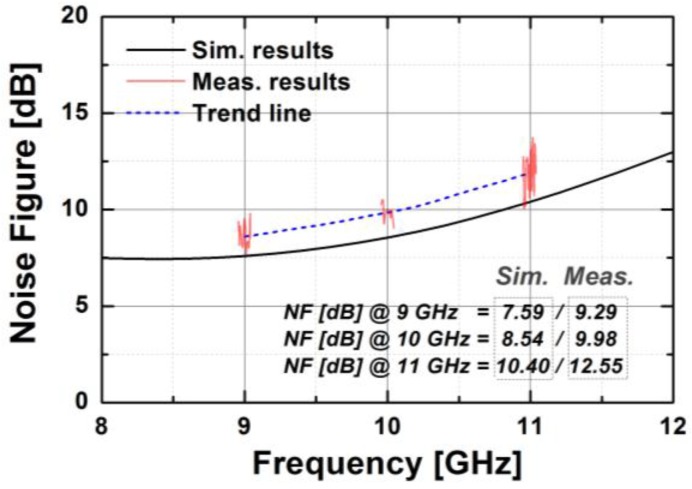
The measured noise figure of the implemented TRM.

**Figure 22:**
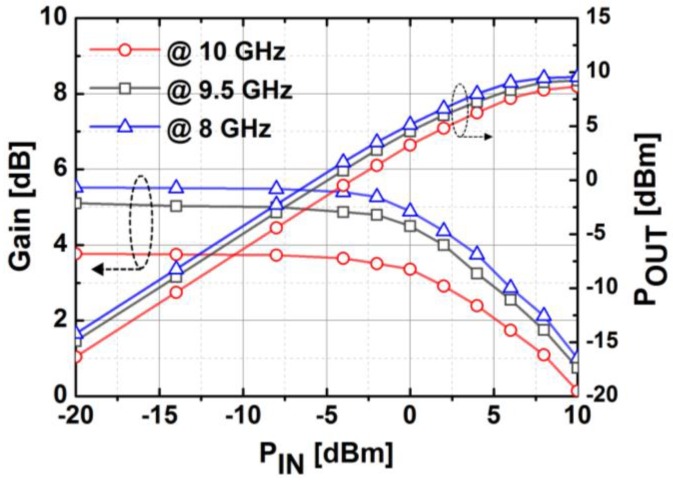
The measured output power and gain of the implemented TRM at different frequencies.

**Figure 23:**
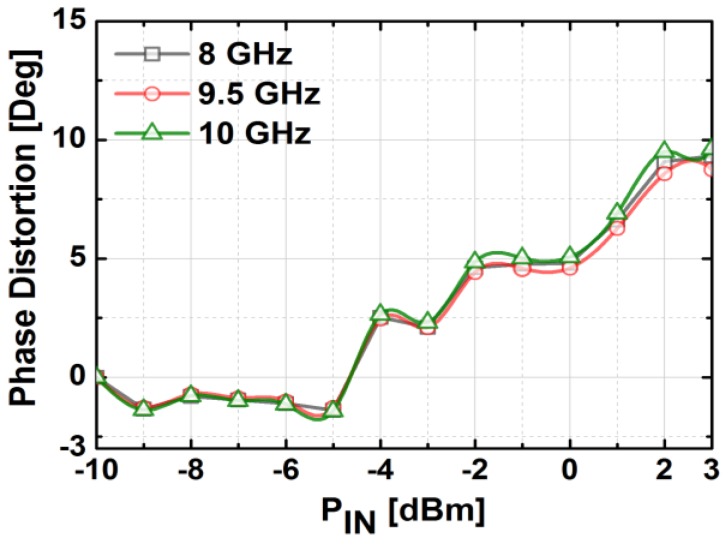
The measured AM/PM conversion of the implemented TRM at different frequencies.

Figure 20 presents the measured S-parameters of the fabricated TRM at the reference state in which both phase shifter and attenuator blocks were set to zero. The transmission gain of the entire chain was around 3.8 dB at 10 GHz, which corresponds well with the simulation. The measured input and output return losses were better than 10 dB, and the isolation between Tx and Rx was higher than 60 dB over the whole X-band. As depicted in Figure 21, the measured NF is about 10 dB at 10 GHz. The measurement results of output power and gain of the TRM at different frequencies are shown in Figure 22. As can be seen, OP1dB was 5.13 dBm at 10 GHz which satisfied the initial specification of the target application. The total DC current consumption was 142 mA with a 1.2 V supply voltage, which was mainly by the BDGAs. We also measured AM/PM conversion with the Keysight N5224A network analyzer by sweeping the input power level as presented in Figure 23 at three different frequencies. The phase distortion (AM/PM conversion) increases sharply when the output signal is saturated with considerable input power.

**Figure 24:**
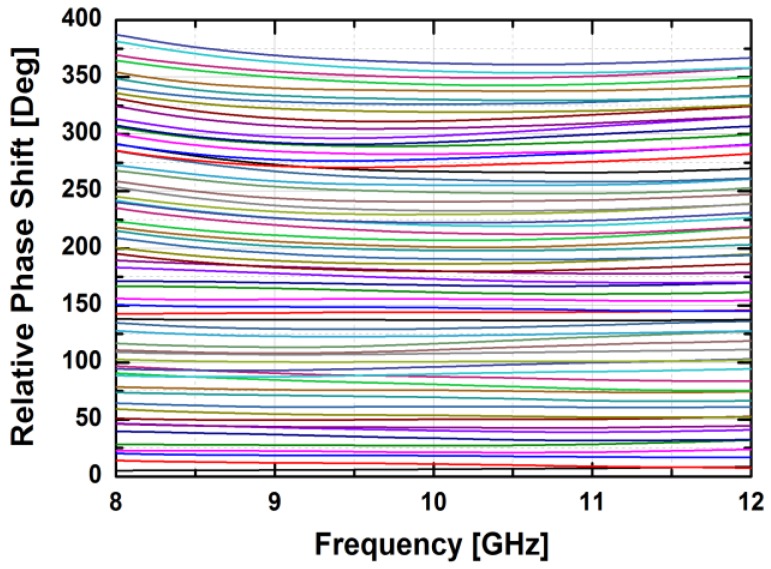
The relative phase shift levels of the fabricated TRM vs. the control bits over frequency.

**Figure 25:**
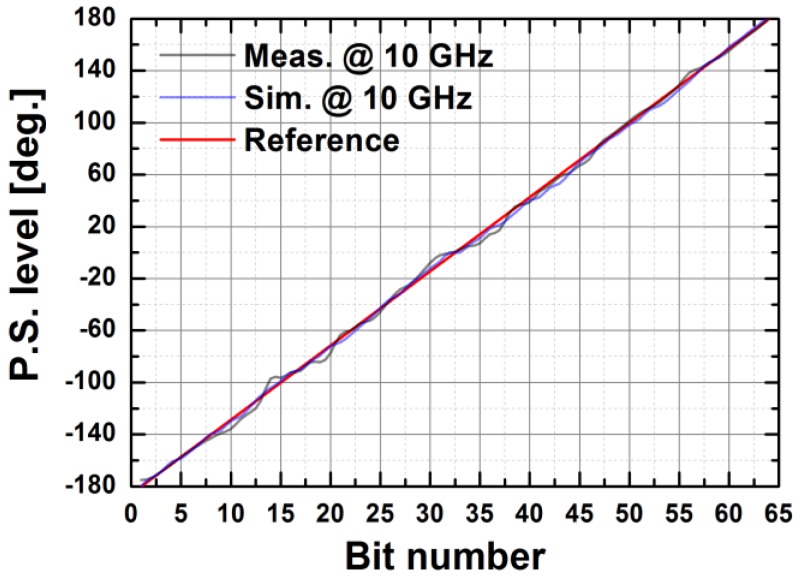
The measured phase shift levels vs. ideal values at 10 GHz as a function the control bits.

**Figure 26:**
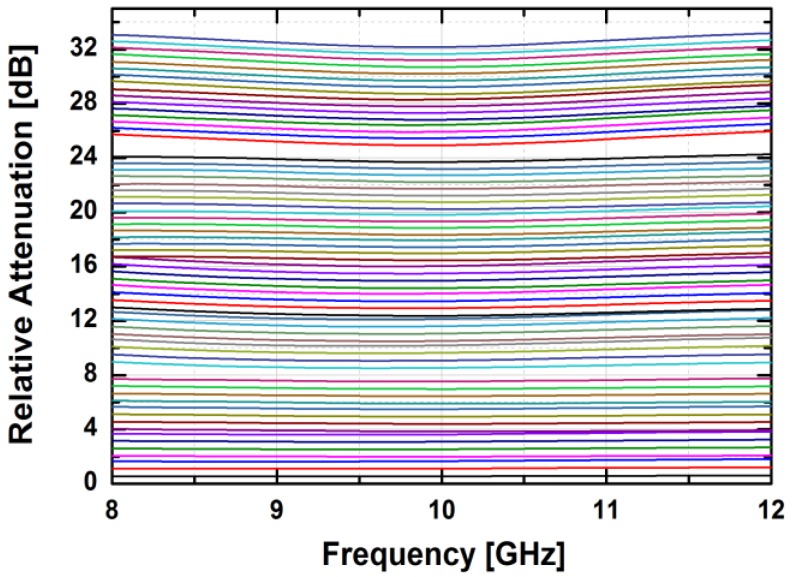
The relative attenuation levels of the TRM vs. the control bits over frequency.

**Figure 27:**
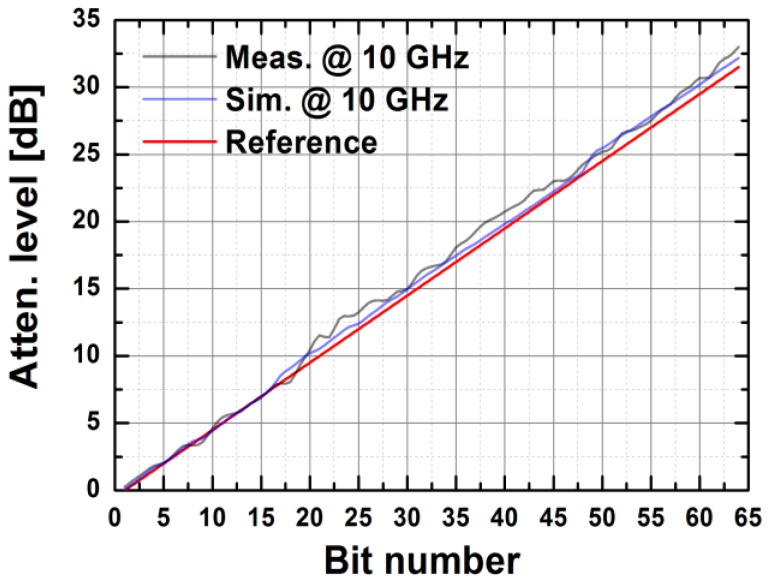
The measured attenuation levels vs. expected levels of the TRM at 10 GHz.

**Figure 28:**
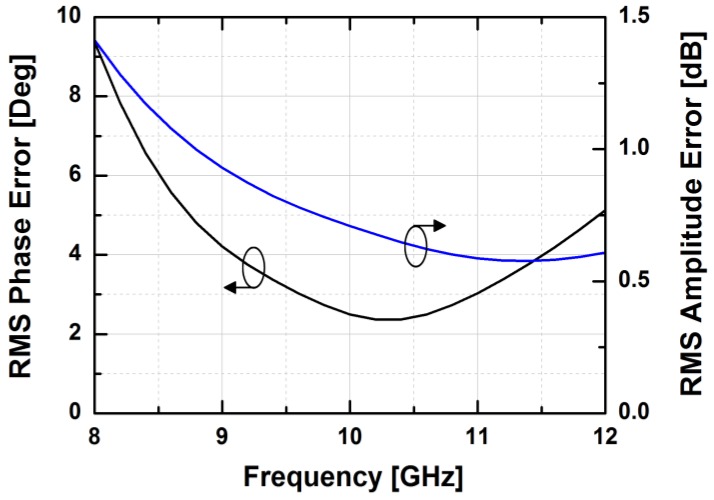
The worst RMS phase and amplitude errors vs. frequency in all of the TRM phase shift states.

**Figure 29:**
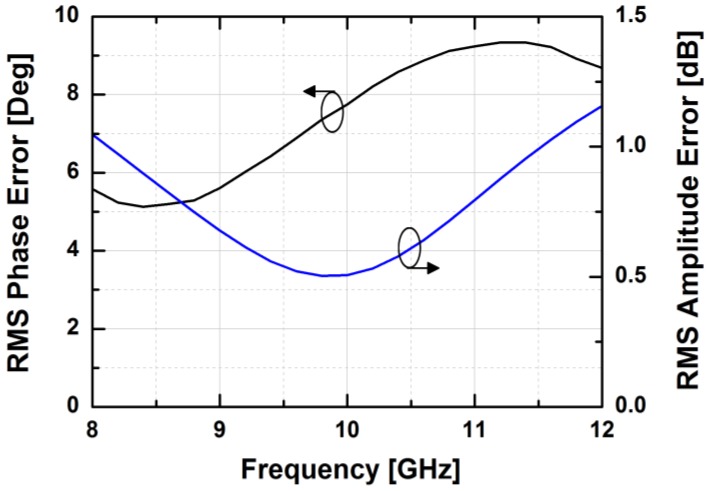
The worst RMS phase and amplitude errors vs. frequency in all of the TRM attenuation states.

**Figure 30:**
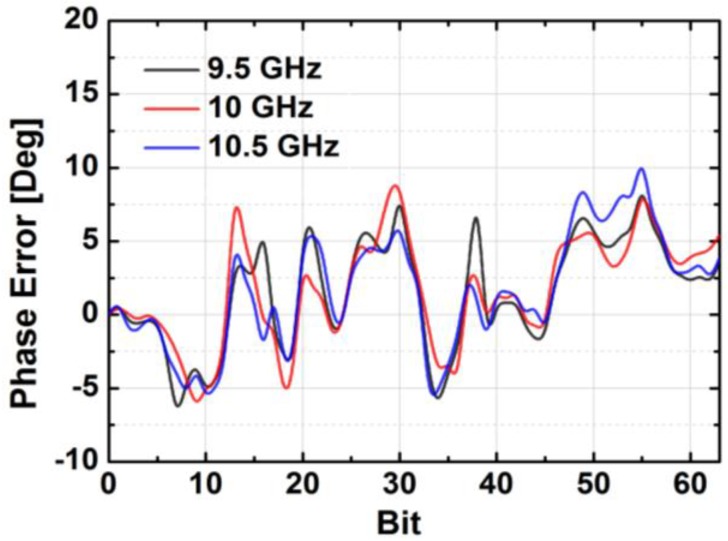
The phase errors of all phase shifting states at different frequencies.

**Figure 31:**
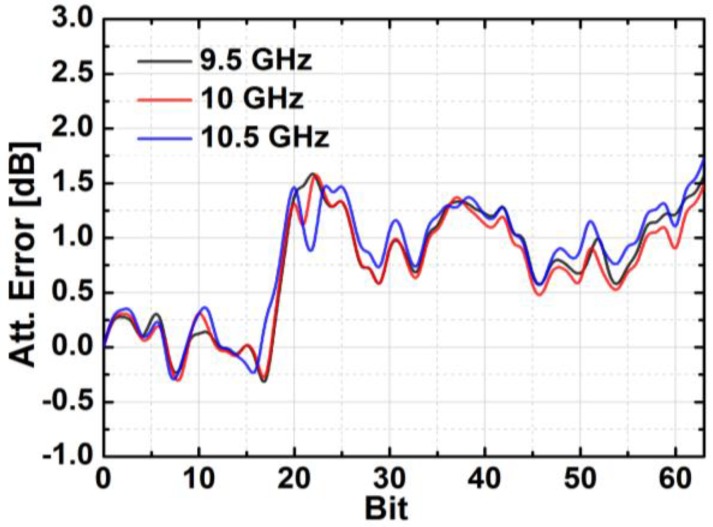
The attenuation errors of all attenuating states at different frequencies.

Figure 24 depicts the measured relative phase response of the fabricated TRM in a total of 64 phase shift states. The system could generate a phase shift ranging from 0° to 360° with a step of 5.625° from 8 to 12 GHz. Figure 25 demonstrates the excellent correlation between the measured phase responses and the expected values at 10 GHz. Figure 26 shows the relative attenuation levels of the fabricated TRM in a total of 64 attenuation states after setting the phase shifter for zero phase shifting. The system could provide an attenuation level of up to 31.5 dB with a step of 0.5 dB over a range of frequencies from 8 to 12 GHz. Figure 27 presents a comparison between the measured amplitude responses and the theoretical values at 10 GHz. To qualitatively examine the phase shifting performances of the fabricated T/R chipset, the RMS phase and amplitude errors in all of the phase shift states are presented. The measured results show that the RMS phase errors were below 4° from 9 to 11 GHz while the RMS amplitude error was less than 0.9 dB from 9 to 11 GHz, as shown in Figure 28. The RMS amplitude errors in all of the attenuation states with the phase shifter block set to zero are shown in Figure 29. We could see the measured RMS amplitude error was around 0.5 dB at 10 GHz while the RMS phase error was below 8° at 9–11 GHz. The phase and attenuation errors between the measured and the expected (ideal) values over all the phase shifting states, and attenuation errors over all the attenuation states at three different frequencies are also presented in Figure 30 and Figure 31, respectively.

Table 5 compares the measured performance of the implemented TRM with that of the recently published works in various device technologies, which demonstrates that the proposed CMOS TRM has the lowest power consumption with comparable performances.

## 4. Conclusions

In this work, we implemented a CMOS-based T/R chipset with a phase coverage of 360° and an LSB of 5.625°, and an attenuation range of 31.5 dB with a step size of 0.5 dB. The double-well body-floating technique was used to effectively alleviate the insertion-loss from the NMOS devices employed in the SPDT and DPDT switches, which also improved their power handling capability. Bi-directional gain amplifiers were distributed along with the unit blocks of the attenuator to provide the desired power gain and to satisfy the output power required for the entire system. Implemented in 65-nm standard CMOS technology with a 1.2 V power supply, the designed TRM is widely appropriate for various low power and low-cost applications.

## Figures and Tables

**Table 1:** Design equations for calculating the *L* and *C* elements of the LP/HP filter networks.

Filter		*L* Element	*C* Element
HP	T-type	$L=\frac{Z_{0}}{\omega \sin(\Delta \varphi /2)}$	$C=\frac{1}{\omega Z_{0}\tan\left(\Delta \varphi /4\right)}$
	π-type	$L=\frac{Z_{0}}{\omega \tan\left(\Delta \varphi /4\right)}$	$C=\frac{1}{Z_{0}\omega \sin(\Delta \varphi /2)}$
LP	T-type	$L=-\frac{Z_{0}}{\omega }\tan\left(\Delta \varphi /4\right)$	$C=-\frac{\sin(\Delta \varphi /2)}{\omega Z_{0}}$
	π-type	$L=-\frac{Z_{0}\sin(\Delta \varphi /2)}{\omega }$	$C=-\frac{\tan\left(\Delta \varphi /4\right)}{\omega Z_{0}}$

**Table 2:** The real values of the *L* and *C* elements used in the design of phase shifter.

Unit Cell	HP Filter		LP Filter	
	*L* (pH)	*C* (fF)	*L* (pH)	*C* (fF)
11.25	80	1000	55	53
22.5	101	624	110	86
45	231	336	224	168
90	1125	790	330	228
180	796	272	796	336

**Table 3:** The parameters of the devices used in the SPDT and DPDT switches (nf: number of fingers, m: multiplication factor).

Device	SPDT	Device	DPDT
*M*_1_, *M*_2_	130 μm/0.06 μm (nf = 26, m = 2)	*M*_1_~*M*_4_	130 μm/0.06 μm (nf = 26, m = 2)
*M*_3_, *M*_4_	20 μm/0.06 μm (nf = 20)	*M*_5_~*M*_8_	20 μm/0.06 μm (nf = 20)
*L*_1_, *L*_2_	610 pH	*L*_1_~*L*_4_	510 pH
*C*_1_~*C*_3_	3.2 pF	*C*_1_~*C*_4_	3.2 pF

**Table 4:** The device parameters used in the attenuator design.

Att. Unit	M1	M2	R1 (Ω)	R2 (Ω)
0.5 dB	40 μm/0.06 μm	4 μm/0.06 μm	6.7	9.12 k
1 dB	40 μm/0.06 μm	4 μm/0.06 μm	15.2	3.04 k
2 dB	40 μm/0.06 μm	4 μm/0.06 μm	21.7	372.4
4 dB	40 μm/0.06 μm	4 μm/0.06 μm	31.1	82.50
8 dB	40 μm/0.06 μm	4 μm/0.06 μm	78.5	33.5
8 dB (16 dB)	40 μm/0.06 μm	4 μm/0.06 μm	81.3	33.5

**Table 5:** Comparison of TRM performance for X-band phased array.

	[7]	[11]	[17]	This Work
Technology	SiGe BiCMOS	SiGe BiCMOS	CMOS 0.18 μm	CMOS 65 nm
Frequency range (GHz)	8–11	8–11	8.5–10	8–10.5
Phase shifter range/step (deg)	360/11.25	360/11.25	360/5.625	360/5.625
Attenuator range/step (dB)	31/1	15.5/0.5	31.5/0.5	31.5/0.5
Insertion gain (dB)	20	17	12	3.7
RMS phase error (deg)	6	6	2	4
RMS amplitude error (dB)	1.5	1	0.25	0.5
OP1dB (dBm)	18	12	11	5.1
NF	9	9	8.5	10
Power consumption (Watt)	1.5	0.8	0.67	0.17
Chip size (mm^2^)	3.5 × 2.4	3.9 × 4.1	4.4 × 2.9	3.92 × 2.44

## References

[1] Forecast International AESA, Other Developments Drive Radar Production. http://www.defense-aerospace.com/article-view/release/99241/aesa,-other-developments-drive-radar-production.html.

[2] Schuh P., Sledzik H., Reber R., Widmer K., Fleckenstein A., Schweizer B., Oppermann M. (2010). T/R module technologies today and future trends. Proceedings of the 40th European Microwave Conference.

[3] Brookner E. Active phase arrays and digital beamforming: amazing breakthroughs and future trends. Proceedings of the 2008 IEEE Radar Conference (IRSI-2007).

[4] XZ1002-BD. 8.5–11.0 GHz GaAs MMIC Core Chip. M/A-COM Tech Asia. https://www.digchip.com/datasheets/3262797-8-5-11-0-ghz-gaas-mmic-core.html.

[5] Product Datasheet: CGY2170YUH: C Band High Gain Low Noise Amplifier. www.ommic.fr/file/download/CGY2178UH_DS_130910.pdf.

[6] Min B., Rebeiz G.M. (2007). A 10–50 GHz CMOS distributed step attenuator with low loss and low phase imbalance. IEEE J. Solid-State Circuits.

[7] Rebeiz G. (2011). Highly dense microwave and millimeter wave phased array T/R modules using CMOS and SiGe RFICs. Proceedings of the IEEE 12th Annual Wireless Microwave Technology Conference.

[8] Dinc T., Zihir S., Gurbuz Y. (2012). SiGe building blocks for on-chip X-band T/R modules. Proceedings of the IEEE 12th Topical Meetings on Silicon Monolithic Integrated Circuits in RF Systems (SiRF).

[9] Jeong J.C., Yom I.B. (2011). X-band high power SiGe BiCMOS multifunction chip for active phased array radars. Electron. Lett..

[10] Patterson C.E., Cressler J.D. (2011). A lightweight organic X-band active receiving phased array with integrated SiGe amplifiers and phase shifters. IEEE Trans. Antennas Propag..

[11] Carosi D., Bettidi A., Nanni A. A mixed-signal X-band SiGe multi-function control MMIC for phased array radar applications. Proceedings of the 39th European Microwave Conference.

[12] Yeo S.K., Chun J.H., Kwon Y.S. (2010). A 3-D X-band T/R module package with an anodized aluminum multilayer substrate for phased array radar applications. IEEE Trans. Adv. Packag..

[13] He J., Fengman L., Dongkai S. (2015). Design and implementation of a 700–2600 MHz RF SiP module for micro base station. Microsyst. Technol..

[14] Carlofelice A.D., Paulis F.D., Fina A., Marcantonio D.U., Orlandi A., Tognolatti P. (2018). Compact and reliable T/R module prototype for advance space active electronically steerable antenna in 3-D LTCC technology. IEEE Trans. Microw. Theory Tech..

[15] Tian G., Li J., Hou F. Design and implementation of a compact 3-D stacked RF front-end module for micro base station. IEEE Trans. Compon. Packag. Manuf. Technol..

[16] Bettidi A., Carosi D., Cetronio A., Corsaro F., Costrini C., Lanzieri C., Marescialli L. (2010). X-band transmit/receive module MMIC chipset on emerging GaN and SiGe technologies. Proceedings of the IEEE Phased Array Systems and Technology Symposium Digest.

[17] Kang D.W., Kim J.G., Min B.W., Rebeiz G.M. (2009). Single and four-element Ka-band transmit/receive phased-array silicon RFICs with 5-bit amplitude and phase control. IEEE Trans. Microw. Theory Tech..

[18] Ku B.H., Kang D.W., Hong S.C. (2011). CMOS integrated circuits for X-band phased array systems. Proceedings of the IEEE 54th International Midwest Symposium on Circuits and Systems (MWSCAS).

[19] Cho M.K., Yoon S.H., Sim S.H., Jeon L., Kim J.G. (2012). CMOS-based bi-directional T/R chipsets for phased array antenna. Proceedings of the IEEE MTT-S International Microwave Symposium Digest (MTT).

[20] Han J.H., Kim J.H., Park J.S., Kim J.G. (2016). A 28 GHz four channel T/R chipset in 65 nm CMOS technology. Proceedings of the IEEE 5th Asia-Pacific Conference on Antennas and Propagation (APCAP).

[21] Lo W.K., Chan W.S., Li C.W., Leung C.K. (2007). Self-phase equalised bidirectional distributed amplifier. Electron. Lett..

[22] Yang J.M., Lai R., Chung Y.H., Nishimoto M., Battung M., Lee W., Kagiwada R. (2004). Compact Ka-band bi-directional amplifier for low-cost electronic scanning array antenna. IEEE Solid-State Circuits.

[23] Min B.W., Rebeiz G.M. (2008). Ka-band low-loss and high-isolation switch design in 0.13 m CMOS. IEEE Trans. Microw. Theory Tech..

[24] Dinc T., Zihir S., Gurbuz Y. (2010). CMOS SPDT T/R switch for X-band, on-chip radar applications. Electron. Lett..

[25] Huang F.J., Kenneth O. (2001). A 0.5-µm CMOS T/R switch for 900-MHz wireless applications. IEEE J. Solid-State Circuits.

[26] Kidwai A.A., Fu C.T., Jensen J.C., Taylor S.S. (2009). A fully integrated ultra-low insertion loss T/R switch for 802.11b/g/n application in 90 nm CMOS process. IEEE J. Solid-State Circuits.

[27] Li Z., Yoon H., Huang F.J. (2003). 5.8-GHz CMOS T/R switches with high and low substrate resistances in a 0.18-µm CMOS process. IEEE Microw. Wirel. Compon. Lett..

[28] Yeh M.C., Tsai Z.M., Liu R.C., Lin K.Y., Chang Y.T., Wang H. (2006). Design and analysis for a miniature CMOS SPDT switch using body-floating technique to improve power performance. IEEE Trans. Microw. Theory Tech..

[29] Li Q., Zhang Y.P., Yeo K.S., Lim W.M. (2008). 16.6- and 28-GHz fully integrated CMOS RF switches with improved body floating. IEEE Trans. Microw. Theory Tech..

[30] Stefano T., Thomas B., Stefan B., Andreas D., Peter K. Electronically steered cognitive weather radar—A technology perspective. Proceedings of the 2017 IEEE Radar Conference.

